# Cerebral apolipoprotein E and amyloid precursor-like protein 1 as risk factors for chronic neurodegeneration after non-traumatic acute brain injury (ABI)

**DOI:** 10.1186/s13054-023-04538-y

**Published:** 2023-06-24

**Authors:** Carlos A. Santacruz, Jean-Louis Vincent, Virginie Imbault, Michael Bruneau, Jacques Creteur, Serge Brimioulle, Raussens Vincent, David Communi, Fabio S. Taccone

**Affiliations:** 1 Department of Intensive Care, Erasme Hospital, Université Libre de Bruxelles, Brussels, Belgium; 2 Department of Intensive and Critical Care Medicine, Fundación Santa Fe de Bogota, Bogota, Colombia; 3 Institut de Recherche Interdisciplinaire en Biologie Humaine et Moléculaire, Université Libre de Bruxelles, Brussels, Belgium; 4 Laboratory for the Structure and Function of Biological Membranes, Center for Structural Biology and Bioinformatics, Université Libre de Bruxelles, Brussels, Belgium; 5 Structural Biology and Bioinformatics Center, Structure and Function of Biological Membranes, Faculty of Science, Université Libre de Bruxelles, Brussels, Belgium

Acute brain injury (ABI) is defined as damage to the brain parenchyma as a result of an acute traumatic or non-traumatic (e.g., subarachnoid hemorrhage [SAH], ischemic stroke, intracerebral hemorrhage [ICH], acute demyelinated encephalopathy [ADE], acute hydrocephalus) injury [1, 2]. Patients with traumatic brain injury (TBI) are at an increased risk of developing chronic neurodegenerative diseases, including Alzheimer's disease (AD) [2], an association thought to be related to several factors, including altered production and clearance of apolipoprotein E (ApoE) and amyloid-β (Aβ) [3, 4]. ApoE is involved in lipid transport and injury repair in the brain. Lipidated ApoE binds to soluble Aβ and facilitates Aβ uptake through cell surface receptors, including low-density lipoprotein receptor (LDLR), LDLR-related protein 1 (LRP1), and heparan sulphate proteoglycan [4]. Aβ is produced by the cleavage of amyloid precursor proteins (APPs) in the cell membrane by b-secretase and g-secretase into a monomeric form with peptides of different lengths such as amyloid beta 1–40 (Aβ_1–40_), and amyloid beta 1–42 (Aβ_1–42_), which is then transformed into oligomeric and fibril forms and is one of the hallmarks of AD [5]. Aβ_1–40_ and Aβ_1–42_ speed up the aggregation kinetics and alter the pattern of spontaneously formed oligomeric species, which are considered the main toxic species [5–8]. Mounting evidence from genetic, pathological, and functional studies has shown that accumulation and aggregation of Aβ in the brain is the result of an imbalance between its production and clearance. The Aβ peptides aggregate as toxic soluble Aβ oligomers, which then form amyloid plaques that injure synapses and ultimately cause neurodegeneration and dementia. After TBI, the concentration of ApoE in the cerebrospinal fluid (CSF) is low, which represents a risk factor for the development of Alzheimer's disease-like disease [9, 10]. Additionally, reduced levels of CSF Aβ_1–42_ accumulation in Aβ plaques, which are the pathological hallmark of AD, can be found in terminal axons within hours of death in up to 30% of patients with TBI [3]. However, data on the associations of ApoE and Aβ in patients with non-traumatic ABI have rarely been reported.

We previously reported decreased CSF expression of proteins related to cholesterol metabolism in patients with ABI [1], which may potentially lead to reduced neuro-steroid production, increased risk of neurodegenerative disease, and worse functional outcomes. In a post hoc analysis of that study, we tested the hypothesis that low ventricular CSF (vCSF) concentrations of ApoE and Aβ-related proteins would also be present early after non-traumatic ABI and may be associated with unfavorable neurological outcome, defined as a Glasgow Outcome Scale score of 1–3 at 3 months after admission. In this post hoc analysis, ApoE (ApoE_elisa_), amyloid beta 1–40 (Aβ_1–40_), and amyloid beta 1–42 (Aβ_1–42_) concentrations were measured in vCSF taken from an external ventricular drain on Days 1–5 after non-traumatic ABI. Control patients had scheduled elective clipping of a non-ruptured aneurysm and CSF was sampled just prior to surgery (Day 1). All samples were volume normalized. The vCSF concentrations of ApoE were measured using a commercial enzyme-linked immunosorbent assay (ELISA) test (Quantikine® ELISA human apolipoprotein E/ApoE Immunoassay, R&D Systems® Minneapolis, MN) according to the manufacturer’s protocol. The vCSF concentrations of Aβ_1–40_ and Aβ_1–42_ were also measured using a commercial ELISA test (human Aβ40 and Aβ42 ELISA kits, Invitrogen, Thermofisher USA) according to the manufacturer’s protocol, with 50 µl/patient (robotic pipette) per sample in duplicate using Aβ_1–40_ and Aβ_1–42_ monoclonal antibody (Biognost®, Germany), respectively. ApoE_elisa_ data were available for 19 patients with non-traumatic ABI (10 SAH, 2 ischemic stroke, 5 ICH, 1 ADE, 1 acute hydrocephalus) and for 5 control patients; data for Aβ_1–40_ were available for 20 patients with non-traumatic ABI (11 SAH, 2 ischemic stroke, 4 ICH, 1 ADE, 2 acute hydrocephalus) and 10 controls; and data for Aβ_1–42_ were available for 23 patients with non-traumatic ABI (SAH, n = 14; IS, n = 2; ICH, n = 5, ADE, n = 1; acute hydrocephaly, n = 1) and 10 controls. We also used data independent acquisition and SWATH mass spectrometry (SWATH-DIA) to measure ApoE (ApoE_swath_), APP, and amyloid precursor-like 1 (APLP1) and 2 (APLP2) as previously described [1]; data using this technique were available from 29 patients with non-traumatic ABI (19 SAH, 3 ischemic stroke, 6 ICH, 1 acute hydrocephalus) and 10 controls. ELISA samples were taken only on Day 1 and SWATH-DIA on days 1–5. We used a Shapiro–Wilk's test to assess distribution and a non-parametric Wilcoxon test to quantify differences in vCSF proteomic expression between the groups. All tests were done using the latest version of R language [11]. The original study [1] was approved by the Erasme Hospital (Brussels, Belgium) Ethics Committee (2014/170–2015/130) and all patients or their next-of-kin signed written informed consent.

Day 1 ApoE_elisa_ concentrations were significantly lower (*p* < 0.01) in patients with non-traumatic ABI than in control patients. vCSF Aβ_1–40_ (*p* < 0.001) and Aβ_1–42_ (*p* < 0.001) concentrations were also lower in the ABI patients (Fig. 1). Day 1–5 ApoE_swath_ protein expressions were significantly lower in patients with non-traumatic ABI than in controls (*p* < 0.0001) (Additional file 1: Table S1). APLP1 values, but not those of APP or APLP2, were also significantly lower in non-traumatic ABI (*p* < 0.0001) throughout the 5-day period (Table S1). There were no differences in ApoE or Aβ proteins in patients with unfavorable and favorable neurological outcomes (data not shown).

**Fig. 1:**
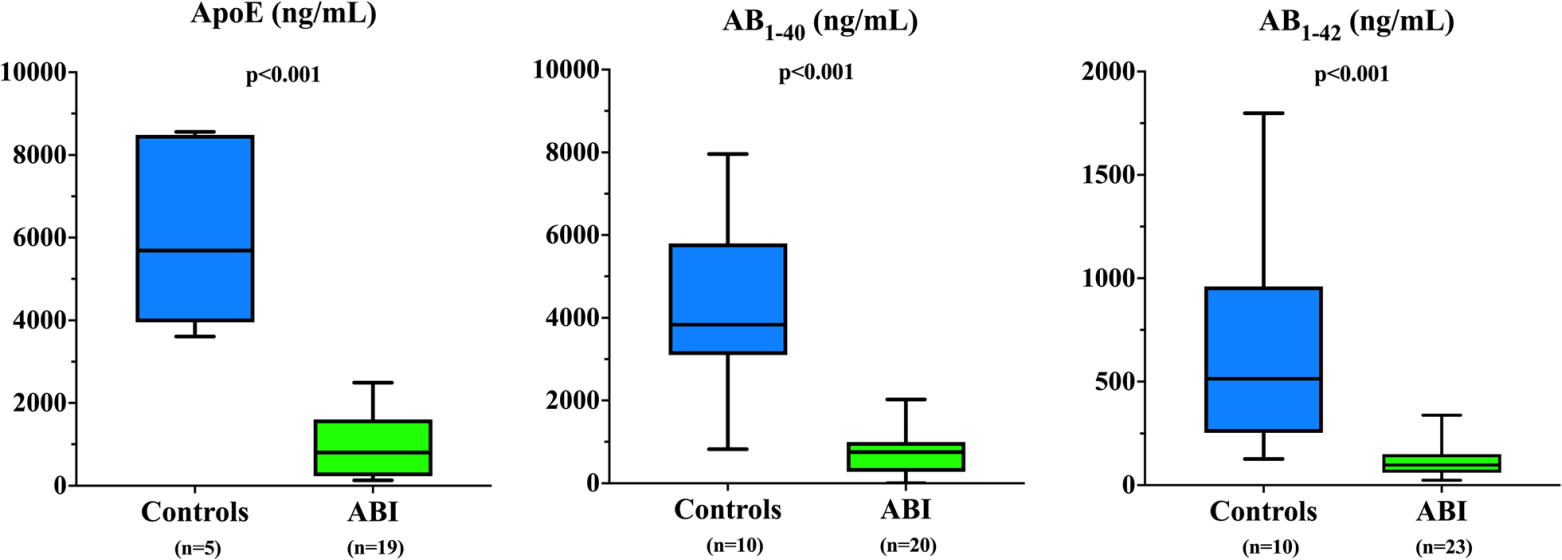
Box plot of results from ELISA testing for ApoE and Aβ-related proteins in the CSF of patients with non-traumatic ABI without ABI (controls). Boxes represent the 25th–75th interquartile ranges with medians (horizontal line); the whiskers represent the minimum and maximum values in the dataset

These findings suggest that specific precursors of neurotoxic Aβ may be expressed in the early phase of non-traumatic ABI, such as that caused by vascular injury after ischemic or hemorrhagic strokes. However, this proteomic pattern in the vCSF was not associated with 90-day neurological outcome, which may be explained by various reasons. First, ABI cannot be entirely responsible for 90-day outcome, as many other factors contribute to functional recovery, such as central nervous system infection, seizures, rehabilitation, and pre-existing brain dysfunction. Second, the GOS is a measure of functional outcome rather than neurocognitive outcome and may not be the most suitable measure for assessing correlations between proteomic profiles and risk of developing neurocognitive disease. Third, not all the analyzed vCSF samples gave an ELISA result, which may have introduced a bias in the results. Lastly, the sample size was too small to allow normalization for age or comorbidities [1].

We acknowledge that this study is more valuable as an exploration of the proteomic profile after non-traumatic ABI rather than to assess its impact on neurological outcome. More studies on the vCSF proteomic profile of patients with ABI are needed to help better understand the cerebral response to an acute injury and the effect of such profiles on neurological outcomes in this setting.

## Supplementary Information

**Additional file 1**.** Supplemental Table S1**. Comparisons of MS/MS swath results between patients without ABI (controls) and those with non-traumatic ABI. ApoE: Apolipoprotein E; AB1:40 amyloid beta 1:40; AB1:42 amyloid beta 1:42; APP: amyloid precursor protein; APLP1: amyloid precursor-like protein 1; APLP2: amyloid precursor-like protein 2. *ompared to Day 1 results for controls. **: MS/MS SWATH-DIA is a measure of abundance with no specific unit of quantification.

## Data Availability

The datasets used and/or analyzed during the current study are available from the corresponding author on reasonable request.
