# Supplementary material for: Cerebral apolipoprotein E and amyloid precursor-like protein 1 as risk factors for chronic neurodegeneration after non-traumatic acute brain injury (ABI)
Source: Crit Care. 2023 Jun 24;27:249. doi: 10.1186/s13054-023-04538-y (PMC10290338; doi:10.1186/s13054-023-04538-y)
Supplement: Supplementary file 1 — Additional file 1. Supplemental Table S1. Comparisons of MS/MS swath results between patients without ABI (controls) and those with non-traumatic ABI. ApoE: Apolipoprotein E; AB1:40 amyloid beta 1:40; AB1:42 amyloid beta 1:42; APP: amyloid precursor protein; APLP1: amyloid precursor-like protein 1; APLP2: amyloid precursor-like protein 2. *ompared to Day 1 results for controls. **: MS/MS SWATH-DIA is a measure of abundance with no specific unit of quantification. [file 13054_2023_4538_MOESM1_ESM.docx]

**Supplementary material**

Santacruz et al.

Table S1. Day 1 to 5 values of ApoE_swath_, APP, APLP1 and 2 in patients with non-traumatic ABI compared to day 1 values in control patients.

| **Protein** | No ABI (controls), median | IQR | Non-traumatic ABI, median | IQR | No ABI (controls) vs non-traumatic ABI  p-value |
| --- | --- | --- | --- | --- | --- |
| **ApoE_swath_ **** |  |  |  |  |  |
| **Day 1** | 1844690 | 1479459-2416584 | 351901 | 230505-516845 | 2.8e-07 |
| **Day 2** | NA | NA | 328077 | 230907-549604 | 4.0e-07* |
| **Day 3** | NA | NA | 369568 | 261162-611302 | 0.002* |
| **Day 4** | NA | NA | 335362 | 195638-711422 | 0.001* |
| **Day 5** | NA | NA | 391622 | 288079-906807 | 0.02* |
| **APP **** |  |  |  |  |  |
| **Day 1** | 176452 | 118515-204647 | 112508 | 88698-132213 | 0.13 |
| **Day 2** | NA | NA | 102189 | 74115-138251 | 0.06* |
| **Day 3** | NA | NA | 110281 | 78929-166872 | 0.41* |
| **Day 4** | NA | NA | 119086 | 89698-154597 | 0.38* |
| **Day 5** | NA | NA | 116205 | 88177-162110 | 0.14* |
| **APLP1 **** |  |  |  |  |  |
| **Day 1** | 814276 | 611522-1387458 | 168162 | 140999-186103 | 5.2e-05 |
| **Day 2** | NA | NA | 192294 | 130872-239048 | 3.2e-08* |
| **Day 3** | NA | NA | 190094 | 126685-253970 | 0.001* |
| **Day 4** | NA | NA | 228170 | 159270-267475 | 0.001* |
| **Day 5** | NA | NA | 192286 | 138915-251777 | 0.05* |
| **APLP2 **** |  |  |  |  |  |
| **Day 1** | 30200 | 21274-35435 | 21928 | 13591-35165 | 0.48 |
| **Day 2** | NA | NA | 30086 | 15966-47771 | 0.78* |
| **Day 3** | NA | NA | 22740 | 15418-38592 | 0.17* |
| **Day 4** | NA | NA | 26373 | 15762-54266 | >0.1* |
| **Day 5** | NA | NA | 30962 | 17041-47006 | >0.1* |

**Supplemental Table S1.** Comparisons of MS/MS swath results between patients without ABI (controls) and those with non-traumatic ABI. ApoE: Apolipoprotein E; AB1:40 amyloid beta 1:40; AB1:42 amyloid beta 1:42; APP: amyloid precursor protein; APLP1: amyloid precursor-like protein 1; APLP2: amyloid precursor-like protein 2. *ompared to Day 1 results for controls. **: MS/MS SWATH-DIA is a measure of abundance with no specific unit of quantification**.**
